# The incidence of venous thromboembolism in patients undergoing arthroscopic anterior cruciate ligament repair: A proposed thromboprophylaxis regimen

**DOI:** 10.5339/qmj.2025.42

**Published:** 2025-06-09

**Authors:** Yousef Al-Khatib, Manoj Kumar, Malak Alwaheed, Bisrat Girma Berhanu, Omran Al-Khatib, Mohamad Askar, Ayaz Lakdawala

**Affiliations:** 1 Trauma and Orthopaedics, Stoke Mandeville Hospital, Aylesbury, UK; 2 Trauma and Orthopaedics, George Eliot Hospital, Nuneaton, UK; 3 Trauma and Orthopaedics, Connolly Hospital, Dublin, Ireland; 4 Department of Education, University Hospitals of Leicester NHS Trust, Leicester, UK; 5 General Surgery, Royal Hampshire County Hospital, Winchester, UK; 6 Faculty of Medicine, Mansoura University, Mansoura, Egypt *Email: yousefal-khatib@outlook.com

**Keywords:** Anterior cruciate ligament reconstruction, venous thromboembolism, arthroscopy, venous thromboembolism prophylaxis

## Abstract

**Introduction:**

Venous thromboembolism (VTE) following anterior cruciate ligament (ACL) arthroscopic reconstructions is reported to occur at a rate of 0.5%–2.2%, with very few studies investigating the use of thromboprophylaxis. This study aims to investigate the incidence of VTE post ACL reconstruction surgery while proposing a thromboprophylaxis regimen.

**Methods:**

A single-center retrospective cross-sectional observational study was conducted over 8 years and 8 months. The primary outcome was the incidence of symptomatic VTE up to 12 weeks post-operatively. Secondary outcome measures were the rate of major bleeding incidents, wound infections, and delayed wound healing. Enoxaparin 40 mg subcutaneously once daily and thromboembolic deterrent stockings were given to all patients for 14 days post-operatively. Total anesthetic time, total surgical time, and tourniquet time were also recorded. Only patients who underwent arthroscopic ACL reconstruction were included, with all conservatively managed patients being excluded.

**Results:**

A total of 155 patients were identified, and none had a symptomatic VTE up to 12 weeks post-operatively. None of the patients experienced delayed wound healing, wound infections, or major bleeding incidences up to 12 weeks post-operatively. Average total anesthetic time was 145 (±24.8) minutes, average total surgical time was 122 (±25.3) minutes, and average Tourniquet time was 82.1 (±23.8) minutes.

**Conclusion:**

We demonstrated a 0% rate of clinically symptomatic VTE without complications such as delayed wound healing or major bleeding incidents. This is the only study proposing a combined regimen of both chemical and mechanical thromboprophylaxis after ACL reconstruction. Further research involving larger groups would be required to assess the effectiveness of this approach and to compare the effectiveness of mechanical and chemical thromboprophylaxis after ACL reconstruction.

## Introduction

Arthroscopic anterior cruciate ligament (ACL) reconstruction is a common orthopedic procedure that is frequently performed on relatively young and healthy patient cohorts. They are widely regarded as safe procedures.^1^ There is a small risk of complications associated with knee arthroscopies and ligament reconstruction. Those complications include the risk of deep vein thrombosis (DVT) and pulmonary embolism. Although some studies report low incidences of symptomatic DVTs post-procedure, some patients may have asymptomatic DVTs. The percentage of DVTs has been reported to be as high as 17.9% in the literature.^2^ The risk of such complications increases with patient-specific risk factors such as smoking and obesity and with other factors such as a prolonged tourniquet and anesthetic time during the procedure.^3^

Despite the statistics, post-operative venous thromboembolism (VTE) prophylaxis following ACL reconstruction remains a nonstandard practice, and decisions regarding prophylaxis are primarily dependent on the operating surgeon.

The NICE guidelines recommend 2 weeks of prophylactic enoxaparin following lower limb orthopedic procedures with a total anesthetic time of more than 90 minutes.^4^ As ACL reconstructions can be lengthy procedures^5^ and may therefore require prolonged anesthesia, we propose a standard regimen for VTE prophylaxis. This regimen consists of 14 days of prophylactic enoxaparin and the use of thromboembolic deterrent stockings (TEDS) post ACL reconstruction procedures. Through this study, we will evaluate and report the incidence of symptomatic VTEs post ACL reconstruction using 14 days of chemical prophylaxis with enoxaparin along with mechanical prophylaxis with TEDS.

## Methods

A single center retrospective cross-sectional observational study was carried out to include all patients who underwent ACL reconstruction at a district general hospital in the United Kingdom (George Eliot Hospital National Health Service Trust) from March 26, 2015, to November 9, 2023. The primary outcome measure was the incidence of symptomatic DVT up to 12 weeks post-operatively, with secondary outcomes being wound-related complications or any bleeding incidents up to 12 weeks post-operatively. Factors such as age, gender, ACL graft type, associated meniscal injury (as well as site), revision status, total anesthetic time, total surgical time, tourniquet time, and post-operative mobility (with/without a brace) were recorded. Patients who were conservatively managed were excluded from this study.

All patients received 14 days of enoxaparin 40 mg, administered subcutaneously once daily, following their procedure for VTE prophylaxis, in addition to TEDS. All patients were routinely reviewed in the outpatient clinic at 2 and 12 weeks. The records of all patients were reviewed for the incidence of VTE. All patients were specifically asked at their 3-month review point if they had undergone any VTE investigations or treatments, to identify any patients who might have had a VTE in between clinic appointments or in other hospitals.

Clinic letters as well as operation notes were used to record the outcome measures for each patient. Microsoft Excel was then used to calculate both the average and standard deviation for tourniquet time, as well as surgical and total anesthetic time. Microsoft Excel was also used to calculate the average and range of values for patients’ ages.

All patients who underwent an anteromedial ACL reconstruction with three different graft types were included. Four-strand hamstring grafts, hamstring allograft, or ligament augmentation and reconstruction system (LARS) were used. Patients were in a supine position, and graft harvesting was the first step, except for those who used LARS or a hamstring allograft. Grafts were harvested through a 2 cm posteromedial incision and using the Arthrex minimally invasive graft harvesting set to harvest both the gracilis and semitendinosus tendons. This was followed by femoral tunnel preparation via anteromedial and anterolateral ports after a diagnostic arthroscopy was performed. Tibial tunnel preparation was then performed, followed by graft passage and fixation.

All Patients underwent a standardized post-operative physiotherapy program for 12 months. Patients who had a brace for 6 weeks post-operatively were restricted to a range of motion of 0–90°. Both patients with and without the brace underwent the same 12-month physiotherapy regime, which included quadricep and hamstring strengthening exercises. Approval from our local clinical effectiveness department was sought for the collection and analysis of this data (ID: 16471147).

## Results

One hundred and fifty-five consecutive patients were identified, and none of them had symptomatic DVT up to 12 weeks post-operatively. No patients had delayed wound healing or wound infections, with zero incidents of bleeding up to 12 weeks post-operatively.

**Figure 1:**
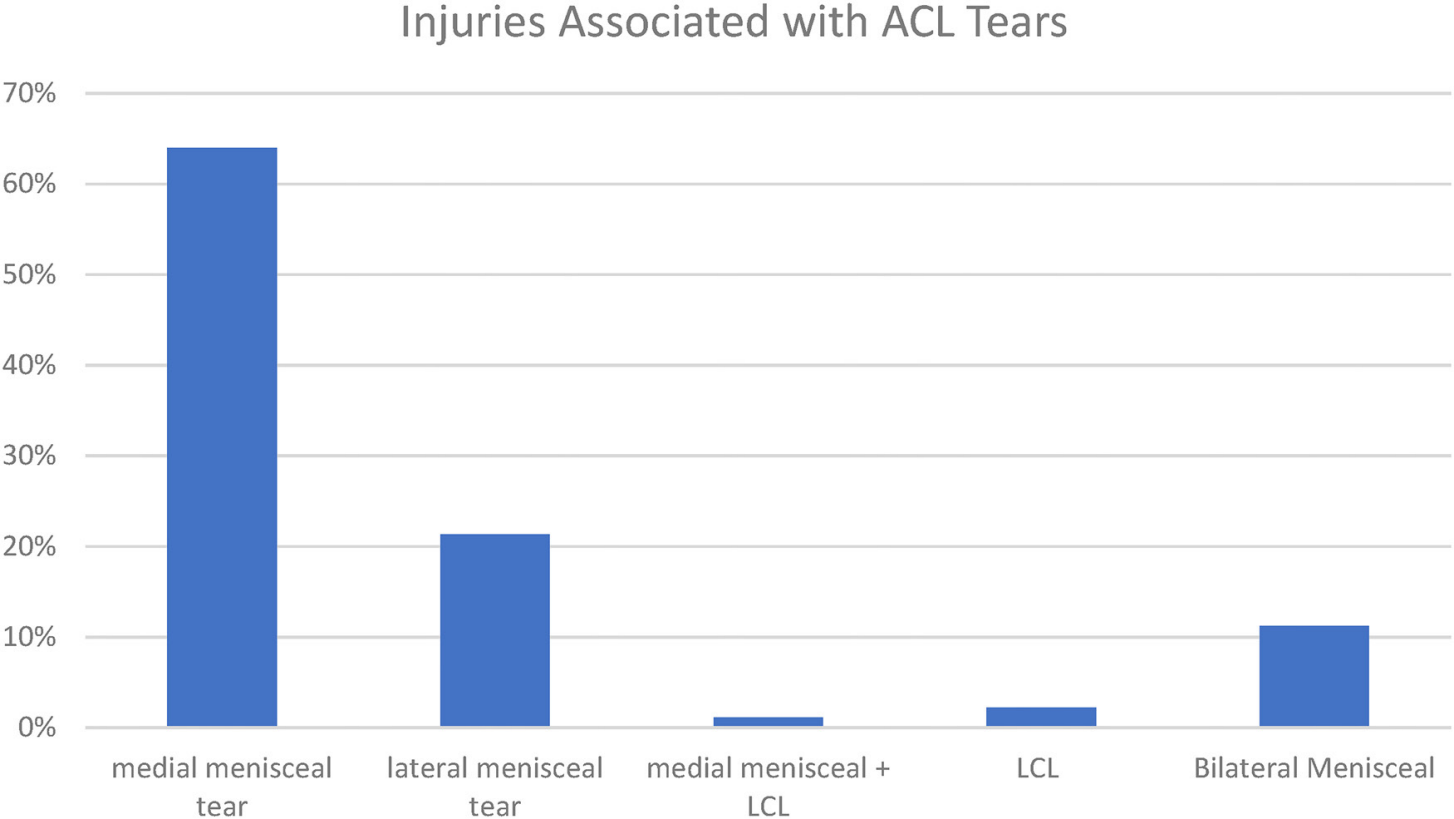
Injuries associated with ACL tears. Data is presented as percentages.

One hundred and forty-five Patients had four-strand hamstring grafts (93.5%), eight (5.2%) had LARS, and two (1.3%) had a hamstring allograft (Table 1). Of the 155 patients, 89 (57.4%) had an associated injury, of which 19 (21.3%) were lateral meniscal tears, 57 (64%) were medial, and 10 (11.2%) were both medial and lateral meniscal injuries. One patient (1.1%) had a medial meniscal as well as lateral collateral ligament (LCL) injury, and two patients (2.2%) had isolated LCL injuries (Figure 1). Meniscal trimming was performed in 34 patients, while repair was performed in 54 patients. 18 out of 155 (11.6%) were revision ACL reconstruction procedures.

All patients underwent day-case procedures and were mobilized on the first postoperative day. 54 (34.8%) Patients were mobilized with a hinge knee brace, while 101 (65.2%) patients were mobilized without a brace post-operatively.

Average total anesthetic time was 145 (±24.8) minutes, average total surgical time was 122 (±25.3) minutes, and average Tourniquet time was 82.1 (±23.8) minutes.

The average patient age was 27.1 years (range = 18–49); 125 (80.6%) were male, and 30 (19.4%) were female patients. Seven out of 155 patients (4.5%) had a documented history of smoking. There were 93 (60%) patients who were active (regularly engaged in sporting activities) or had an active job such as construction or building. Sixty-two (40%) patients were not active and had desk-based jobs and underwent conservative management with physiotherapy, which was ineffective.

## Discussion

This review of patients reveals a 0% incidence of clinically detectable VTE in our patient sample, which is lower than the 0.5%–2.2% in the published literature following ACL reconstruction surgery.^6–9^ A large retrospective analysis of 16,558 patients undergoing ACL reconstruction surgery database suggests a 0.53% risk of VTE, with no further information available on the use of mechanical and chemical thromboprophylaxis in this study.^8^

Age, smoking, high tibial osteotomy, concurrent posterior cruciate ligament (PCL) repair, presence of an active wound infection, and inpatient surgery are risks reported in the literature to be associated with VTE post-ACL reconstruction surgery.^6,10^ None of our patients underwent PCL repair, a high tibial osteotomy, or inpatient surgeries. Only 7 out of 155 patients (4.5%) in this study were smokers.

Yazdi et al. conducted a study investigating the use of enoxaparin versus aspirin as chemical thromboprophylaxis post-ACL surgery. They found no statistical difference in VTE incidence in both the aspirin and enoxaparin groups.^11^ There was no mention of mechanical prophylaxis or TEDS in the study. Our study investigates the efficacy of a 14-day regimen comprising both chemical and mechanical thromboprophylaxis. Yazdi et al. reported a VTE incidence of 1.09% with aspirin and 1.63% with enoxaparin as thromboprophylaxis.^11^ We report a 0% incidence of VTE using TEDS and enoxaparin as a thromboprophylaxis regimen. This finding prompts further research comparing the difference in efficacy of mechanical and chemical thromboprophylaxis when combined or used separately.

The majority of current literature regarding VTE prophylaxis in ACL reconstruction surgery does not detail the precise thromboprophylaxis regimen used. They mostly focus on chemical thromboprophylaxis without mentioning mechanical thromboprophylaxis.^11,12^

Tourniquet use is reported to be associated with an increased risk of asymptomatic DVTs.^13^ Another study reported that a tourniquet time of over 90 minutes was associated with an increased risk of DVT following knee arthroscopy.^3^ The average tourniquet time in this study was 82.1 minutes (±23.8). Prolonged surgical time is also associated with an increased VTE Incidence.^14^ Surgical time was 122 minutes (±25.8) in our study. Increased anesthetic time was another factor associated with a higher risk of VTE.^15^

With regards to limitations, the design of this study did not involve routine ultrasound scanning of all patients postoperatively, so it is not possible to comment on the incidence of asymptomatic DVT in this patient group. However, the significance of clinically undetectable DVTs remains controversial. The study also did not examine the patients’ risk factors for VTE at baseline, nor did it assess other medical comorbidities. The major limitation of this study is the limited sample size, as VTE is a relatively rare complication following ACL surgery.^10–15^

There are several facets to the regimen described, and understandably, concerns may arise regarding potential cost implications. It is only possible to discuss the cost based on the UK healthcare system, specifically the National Health Service. The total cost for the duration of treatment is £146.36 for a 14-day course of enoxaparin post-operatively, but this does not include the cost of TEDS used. There is significant morbidity associated with VTE, along with the potential for an increased/additional length of stay. The exact cost is difficult to determine, but considering the increased bed stay alone, which is approximately £400 per day, this is a minimal cost. This does not account for the cost of ongoing treatment for VTE, such as treatment with Factor Xa inhibitors, which costs a minimum of £180 for a 3-month course of treatment.

Overall, there is a paucity of good evidence regarding the risks and benefits of mechanical and chemical prophylaxis in ACL reconstruction surgery. We believe this is the first study to propose a specific regimen for the perioperative mechanical and chemical VTE prophylaxis in patients undergoing ACL reconstruction surgery with robust patient numbers and documented regimen efficacy. It is also in line with the National Institute for Health and Care Excellence guidelines for VTE thromboprophylaxis in lower limb surgery.^4^

## Conclusion

This study proposes a combined chemical and mechanical thromboprophylaxis regimen post ACL reconstruction to reduce the incidence of symptomatic VTE. We demonstrated a 0% incidence of clinically symptomatic VTE; however, routine postoperative ultrasound imaging is needed to confirm the absence of VTE in asymptomatic patients. Nonetheless, we have demonstrated a reduction in the incidence of symptomatic VTE post ACL reconstruction compared to published literature. Further studies with larger sample sizes would be necessary to evaluate this regime’s efficacy more thoroughly, as well as to compare the efficacy of mechanical and chemical thromboprophylaxis post ACL reconstruction.

## Figures and Tables

**Table 1.** ACL graft type.

**Graft type**	**Number of patients**	**Percentage (%)**
Four-strand hamstring	145	93.5
LARS	8	5.2
Hamstring allograft	2	1.3

LARS: ligament augmentation and reconstruction system.
